# Effects of Insulin on Porcine Neonatal Sertoli Cell Responsiveness to FSH In Vitro

**DOI:** 10.3390/jcm8060809

**Published:** 2019-06-06

**Authors:** Rossella Cannarella, Iva Arato, Rosita A. Condorelli, Laura M. Mongioì, Cinzia Lilli, Catia Bellucci, Sandro La Vignera, Giovanni Luca, Francesca Mancuso, Aldo E. Calogero

**Affiliations:** 1 Department of Clinical and Experimental Medicine, University of Catania, 95123 Catania, Italy; rosita.condorelli@unict.it (R.A.C.); lauramongioi@hotmail.it (L.M.M.); sandrolavignera@unict.it (S.L.V.); acaloger@unict.it (A.E.C.); 2 Department of Experimental Medicine, University of Perugia, 06132 Perugia, Italy; iva.arato@libero.it (I.A.); cinzia.lilli@unipg.it (C.L.); catia.bellucci@unipg.it (C.B.); giovanni.luca@unipg.it (G.L.); francesca.mancuso@unipg.it (F.M.)

**Keywords:** FSH, insulin, Sertoli cells, AMH, inhibin B

## Abstract

There is ongoing debate as to whether the decline of sperm production in recent times may be related to a parallel increase in the rate of obesity and diabetes. Lower anti-Müllerian hormone (AMH) and inhibin B secretion have been observed in young hyperinsulinemic patients compared to healthy controls, suggesting a Sertoli cell (SC) dysfunction. The pathophysiological mechanisms underlying SC dysfunction in these patients are poorly understood. To the best of our knowledge, no evidence is available on the effects of insulin on SC function. Therefore, this study was undertaken to assess the effects of insulin on basal and follicle-stimulating hormone (FSH)-stimulated SC function in vitro. To accomplish this, we evaluated the expression of *AMH*, *inhibin B* and *FSHR* genes, the secretion of AMH and inhibin B and the phosphorylation of AKT473 and SC proliferation on neonatal porcine SC after incubation with FSH and/or insulin. We found that similar to FSH, the expression and secretion of AMH is suppressed by insulin. Co-incubation with FSH and insulin decreased AMH secretion significantly more than with FSH alone. Insulin had no effect on the expression and secretion of the *inhibin B* gene, but co-incubation with FSH and insulin had a lower effect on inhibin B secretion than that found with FSH alone. FSH and/or insulin increased AKT473 phosphorylation and SC proliferation. In conclusion, the results of this study showed that insulin modulates SC function. We hypothesize that hyperinsulinemia may therefore influence testicular function even before puberty begins. Therefore, particular care should be taken to avoid the onset of hyperinsulinemia in children to prevent a future deleterious effect on fertility.

## 1. Introduction

Sertoli cells (SCs), the only somatic constituent of the testicular seminiferous epithelium, are mainly involved in supporting spermatogenesis. In particular, they provide structural support to germ cells (GCs), constitute the blood–testicular barrier, assist the movement of GCs within the seminiferous epithelium and guide the maturation of GCs through secretion products. Notably, they support a finite number of GCs [1,2].

Anti-Müllerian hormone (AMH) and inhibin B are dimeric glycoproteins secreted by SCs; both of these hormones belong to the transforming growth factor-β superfamily. Immature and actively proliferating SCs may be found in the testis until puberty. At this stage, the testis is mainly made up of AMH-secreting SCs. The overall number of SCs is known to impact testicular volume. The amount of AMH secreted reflects SC immaturity. When puberty begins, SCs pass to a state of maturity and quiescence. As a result, serum AMH levels decrease while those of inhibin B increase in a follicle-stimulating hormone (FSH)-dependent manner. Concomitant with the onset of spermatogenesis, the testicular volume increases and GCs become the predominant testicular component (for review, see [3,4]).

FSH acts through a G-protein-coupled (GPCR) receptor (FSHR) in SCs. Once stimulated by FSH, as for many GPCRs, the FSHR triggers the Gαs, which activates the adenylate cyclase, resulting in increased intracellular cAMP levels. The latter leads to protein kinase A (PKA) activation [5], which in turn triggers the following major signaling pathways: the phosphoinositide 3-kinase (PI3K)/AKT pathway and the mitogen-activated protein kinase (MAPK) pathway [6,7]. The first is committed to preventing apoptosis, cell proliferation and glucose transport. It is activated in an insulin-like growth factor 1 receptor (IGF1R)-dependent manner [7]. The MAPK pathways, including ERK1/2 and JNK, regulate cell proliferation, differentiation and apoptosis [6]. Both of these pathways are activated by insulin and the insulin-like growth factor (IGF) family [8].

Insulin receptor (INSR) and IGF1R are expressed in SCs [9] and have been found to play a role in adrenal and testis differentiation [6]. The SC-selective knock-out for *INSR* and/or *IGF1R* genes has been shown to have a negative effect on testicular volume in mice [10]. In particular, the SC-INSR knock-out was associated with a 13.6% testis weight decrease, the SC-IGF1R knock-out with a 34.6% decrease and the combined SC-INSR/IGF1R knock-out with a 72.4% testis weight reduction [10], thus suggesting a role for both receptors in SC proliferation.

There is ongoing debate as to whether the decline of sperm production in recent times [11] is related to the parallel increase in the rate of obesity and diabetes [12]. Some evidence points to a possible negative impact of hyperinsulinemia on SC function before puberty, since lower AMH and inhibin B levels have been found in young obese patients compared to normal weight controls [13,14,15]. However, studies on the possible mechanism(s) are lacking and, therefore, no data are available to date on the effect of insulin on SC function.

As cultures of SCs from pre-pubertal porcine testes have been developed to reproduce an in vitro reliable prototype of pre-pubertal human testicular tissue [16], the purpose of this study was to evaluate the effects of insulin on both basal and FSH-stimulated SC function in this model to better understand the rationale of the results reported in humans [13,14,15]. To accomplish this, we evaluated the expression of *AMH*, *inhibin B* and *FSHR* genes, the secretion of AMH and inhibin B, the phosphorylation of AKT473 and the proliferation of SC after incubation with insulin.

## 2. Experimental Section

### 2.1. Ethics Statement

This study was conducted in strict compliance with the Guide for the Care and Use of Laboratory Animals of the National Institutes of Health and Perugia University Animal Care. The protocol was approved by the internal Institutional Ethic Committee (Ministry of Health authorization n. 971/2015-PR, 9/14/2015).

### 2.2. Sertoli Cell Isolation, Culture, Characterization and Function

SCs, obtained from neonatal prepubertal Large White pigs, 7–15 days of age, were isolated according to established methods, with slight modifications [17]. Briefly, after removing the fibrous capsule, the testes were finely chopped and digested twice enzymatically with a mixed solution of trypsin and deoxyribonuclease I (DNase I) in Hanks’ balanced salt solution (HBSS; Merck KGaA, Darmstadt, Germany) and collagenase P (Roche Diagnostics S.p.A., Monza, Italy). The tissue pellet was centrifuged through a 500 μm pore stainless steel mesh. It was then re-suspended in glycine to eliminate residual Leydig and peritubular cells [18]. The resulting pellet was collected and maintained in HAM’s F12 medium (Euroclone, Milan, Italy), supplemented with 0.166 nmol l−1 retinoic acid, (Sigma-Aldrich, Darmstadt, Germany) and 5 mL per 500 mL insulin-transferrin-selenium (Becton Dickinson cat. no. 354352; Franklin Lakes, NJ, USA) in 95% air/5% CO_2_ at 37 °C. After 3 days in culture, the purity and the functional competence of SC monolayers were determined according to previously established methods [16].

### 2.3. Culture and Treatment

When SC monolayers were confluent (after 3 days of culture), they were incubated for 48 h as follows: (1) placebo; (2) 100 nM highly purified urofollitropin (hpFSH) (Fostimon^®^, IBSA Farmaceutici Srl, Rome, Italy); (3) 100 nM recombinant insulin (rInsulin) (Humalog, Eli Lilly Srl, Florence, Italy); (4) 100 nM hpFSH and 100 nM rInsulin.

### 2.4. RT-PCR Analysis

Total RNA was extracted with Trizol^®^ Reagent (Life Techologies, Waltham, MA, USA) according to the manufacturer’s instructions. RNA concentration and purity were determined using Biophotometer Eppendorf. cDNA reverse transcription was carried out for each sample using a cDNA synthesis kit (Thermo Scientific Maxima First Strand cDNA Synthesis Kit for RT-qPCR), according to the manufacturer’s instruction.

The qPCR was performed using 50 ng of the cDNA prepared by RT and a SYBR Green Master Mix (Stratagene, Amsterdam, The Netherlands) (Agilent Technology), using the following primers: AMH, forward primers 5′-GCGAACTTAGCGTGGACCTG-3′, reverse primers 5′-CTTGGCAGTTGTTGGCTTGATATG-3′; inhibin B, forward primers 5′-TGGCTGGAGTGACTGGAT -3′, reverse primers 5′-CCGTGTGGAAGGATGAGG-3′; FSHR forward primers 5′-TTTCACAGTCGCCCTCTTTCCC-3′, reverse primers 5′-TGAGTATAGCAGCCACAGATGACC-3′; actin, forward primers 5′-ATGGTGGGTATGGGTCAGAA-3′, reverse primers 5′-CTTCTCCATGTCGTCCCAGT-3′. qPCR was performed in an Mx3000P cycler (Stratagene), using FAM for detection and ROX as the reference dye.

### 2.5. AMH and Inhibin B Secretion Assay

Aliquots of the culture media of treated and untreated SCs were collected and stored at −20 °C for the assessment of AMH (AMH Gen II ELISA, Beckman Coulter, Webster, TX, USA) (intra-assay CV = 3.9%; inter-assay CV = 5.8%) and inhibin B (inhibin B Gen II ELISA, Beckman Coulter, Webster, TX, USA) (intra-assay CV = 2.8%; inter-assay CV = 4.3%) concentrations as previously described [19].

### 2.6. Western Blot (WB) Analysis

At the end of the incubation period, total cell lysates were collected in a radioimmunoprecipitation assay (RIPA) lysis buffer (Santa Cruz Biotechnology Inc., Santa Cruz, CA, USA). The mixture was centrifuged at 1000× *g* (Eppendorf, Hauppauge, NY, USA) for 10 min, the supernatant was collected and total protein content was measured by the Bradford method [20]. Sample aliquots were stored at −20 °C for Western blot (WB) analysis. The cell extracts were separated by 4–12% SDS-PAGE and equal amounts of protein (70 μg protein/lane) were run and blotted on nitrocellulose membranes (BioRad, Hercules, CA, USA). The membranes were incubated overnight in a buffer containing 10 mM TRIS, 0.5 M NaCl, 1% (*v/v*) Tween 20 (Sigma-Aldrich), rabbit anti-phospho-AKT (Ser473) (dilution factor 1:1000) (Cell Signaling, Danvers, MA, USA), rabbit anti-AKT (dilution factor 1:1000) (Cell Signaling) and mouse anti- Glyceraldehyde 3-phosphate dehydrogenase (GADPH) (6C5):sc-32233 (dilution factor 1:200) (Santa Cruz, Biotechnology, CA, USA) primary antibodies. Primary antibody binding was then detected by incubating the membranes for an additional 60 min in a buffer containing horseradish peroxidase conjugated anti-rabbit (Sigma-Aldrich) (dilution factor, 1:5000) and/or anti-mouse (Santa Cruz Biotechnology Inc.) (dilution factor, 1:5000) IgG secondary antibodies. The bands were detected by enhanced chemiluminescence.

### 2.7. Cell Number and Proliferation

After reaching 50 to 60% confluence, cells were incubated with 0.1 µg/mL colcemid (Sigma-Aldrich, St. Louis, MO, USA) for 3 h [21]. Afterwards they were washed with phosphate buffer (PBS, Lonza, Basel, Switzerland) before the evaluation of the proliferation. For cell proliferation assay, SCs were incubated with 1 µM 5(6)-carboxyfluorescein diacetate N-succinmidyl ester (CFSE) (Sigma-Aldrich, MO, USA) in PBS for 8 min, and then washed with HBSS medium (Lonza, Basel, Switzerland) three times. CFSE-labeled SCs were then cultured at 37 °C and incubated in 5% CO_2_ for 48 h with hpFSH (100 nM) and/or insulin (100 nM). At the end of the stimulation assay, cells were washed with PBS, harvested by tripsinization and counted using an Automated Cell Counter (Invitrogen, Carlsbad, CA, USA) before flow cytometer analysis [22]. Data acquisition was performed on 20,000 events per tube based on a total (gated alive cells) count of forward and side light scatter at approximately 200–300 events per second on a BD FACS ortflow cytometer (BD Biosciences, Franklin Lakes, NJ, USA), analyzed using FACS Diva software (4.0 BD Biosciences, Franklin Lakes, NJ, USA) and gated on appropriate controls in the different cell populations.

### 2.8. Statistical Analysis

Results are shown as the mean ± SEM of three independent experiments, each one performed in triplicate. The average value from the triplicates of each cell culture was used for the statistical analysis. Data were analyzed for statistical significance by one-way ANOVA, followed by the Tukey post-hoc test using SPSS 22.0 for Windows (SPSS Inc., Chicago, IL, USA). A statistically significant difference was accepted when the *p* value was lower than 0.05.

## 3. Results

### 3.1. RT-PCR Analysis

**Figure 1:**
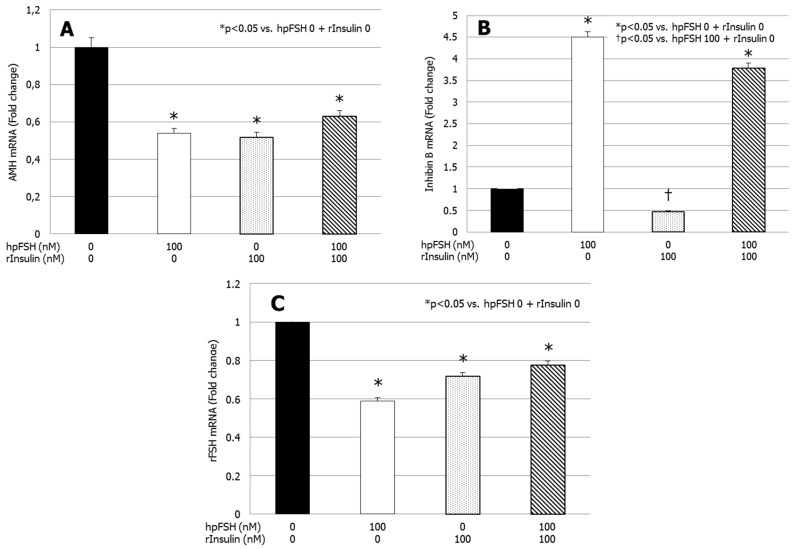
RT-PCR analysis of (**A**) *anti-Müllerian hormone* (*AMH*), (**B**) *inhibin B* and (**C**) *FSHR* gene expression. Data represent the mean ± SEM of three independent experiments, each performed in triplicate. * *p* < 0.05 vs. control; † *p* < 0.05 vs. highly purified urofollitropin (hpFSH) (one-way ANOVA).

Compared with the untreated control, *AMH* gene expression was significantly downregulated by hpFSH, rInsulin and hpFSH plus rInsulin (*p* < 0.05 vs. control) (Figure 1A). Differences in the mRNA levels of AMH were −46.1, −48.1 and −37.0%, respectively.

Compared with the untreated control, *inhibin B* gene expression was significantly upregulated by hpFSH. The co-incubation with hpFSH plus rInsulin did not significantly affect the stimulatory effect of hpFSH alone (Figure 1B). Differences in the mRNA of inhibin B were +350% (*p* < 0.0001) with hpFSH and +279% (*p* < 0.0001) with hpFSH plus rInsulin. Incubation with rInsulin did not have a significant effect on inhibin B mRNA levels compared with untreated control. Compared with hpFSH stimulation, *inhibin B* gene expression was significantly downregulated by rInsulin (−89.4%; *p* < 0.0001) and a trend for lower levels was found after incubation with hpFSH plus rInsulin (−15.8%; *p* = 0.05).

Finally, compared with the untreated control, *FSHR* gene expression decreased significantly after incubation with hpFSH and/or rInsulin (*p* < 0.05 vs. control) (Figure 1C). Differences in the mRNA levels of rFSH were −41.1, −28.2 and −22.5%, respectively. The co-incubation with rInsulin did not change the decrease of *FSHR* gene expression significantly.

### 3.2. AMH and Inhibin B Secretion

**Figure 2:**
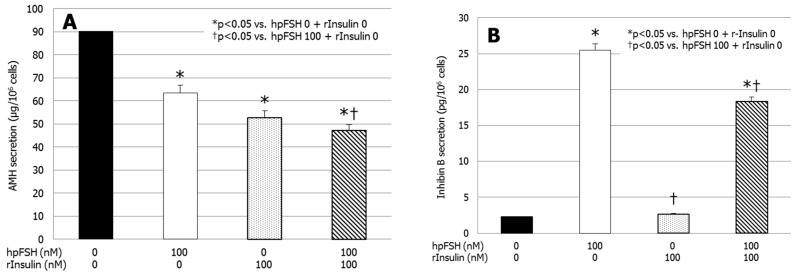
Secretion of (**A**) anti-Müllerian hormone (AMH) and (**B**) inhibin B. Data represent the mean ± SEM of three independent experiments, each performed in triplicate. * *p* < 0.05 vs. control; † *p* < 0.05 vs. hpFSH (one-way ANOVA).

Figure 2A shows a significant decrease of AMH secretion after exposure to hpFSH (63.5 ± 2.7 vs. 90.1 ± 1.4 µg/cell, *p* < 0.0001), rInsulin (52.8 ± 0.6 vs. 90.1 ± 1.4 µg/cell, *p* < 0.0001) and hpFSH plus rInsulin (47.1 ± 0.6 vs. 90.1 ± 4.4 µg/cell, *p* < 0.0001) compared to the untreated control. The co-incubation with hpFSH and rInsulin suppressed AMH secretion significantly compared to that found with hpFSH alone (47.1 ± 0.6 vs. 63.5 ± 2.7 µg/cell, *p* < 0.05). AMH secretion did not differ significantly between hpFSH or rInsulin alone.

Figure 2B shows a significantly higher secretion of inhibin B after hpFSH (25.52 ± 0.52 vs. 2.3 ± 0.8 pg/cell, *p* < 0.0001) and hpFSH plus rInsulin (18.4 ± 0.3 vs. 2.3 ± 0.8 pg/cell, *p* < 0.0001) incubation compared to the untreated control. Treatment with rInsulin did not change inhibin B secretion significantly compared to the untreated control (2.6 ± 1.3 vs. 2.3 ± 0.8 pg/cell, *p* = 0.99). Compared to hpFSH alone, the co-incubation with hpFSH and rInsulin significantly decreased the secretion of inhibin B (18.4 ± 0.3 vs. 25.5 ± 0.5 pg/cell, *p* < 0.0001).

### 3.3. Western Blot (WB) Analysis

**Figure 3:**
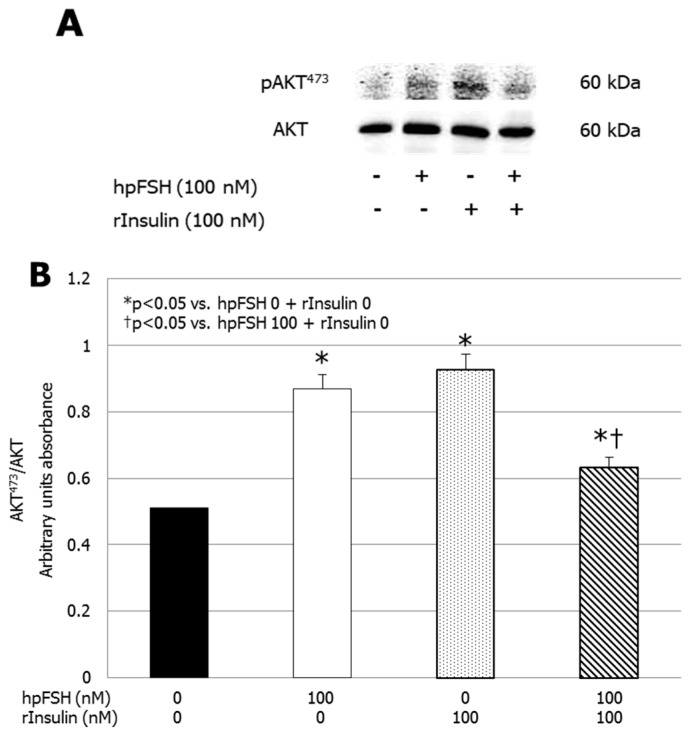
(**A**) Immunoblots and (**B**) densitometric analysis of the protein bands of pAKT473 and AKT of unexposed samples (control) and after incubation with hpFSH, rInsulin or hpFSH plus rInsulin. Data represent the mean ± SEM of three independent experiments, each performed in triplicate. * *p* < 0.05 vs. control; † *p* < 0.05 vs. hpFSH (one-way ANOVA).

WB analysis showed that incubation with hpFSH (0.868 ± 0.07; *p* < 0.001), rInsulin (0.928 ± 0.04; *p* < 0.001) or hpFSH plus rInsulin (0.632 ± 0.04; *p* < 0.01) significantly increased AKT phosphorylation compared to the unexposed control (0.511 ± 0.06). Compared to the hpFSH-treated sample, the levels of AKT phosphorylation after hpFSH plus rInsulin exposure were significantly lower (0.632 ± 0.04 vs. 0.868 ± 0.07, *p* < 0.01) (Figure 3).

### 3.4. Cell Number and Proliferation

**Figure 4:**
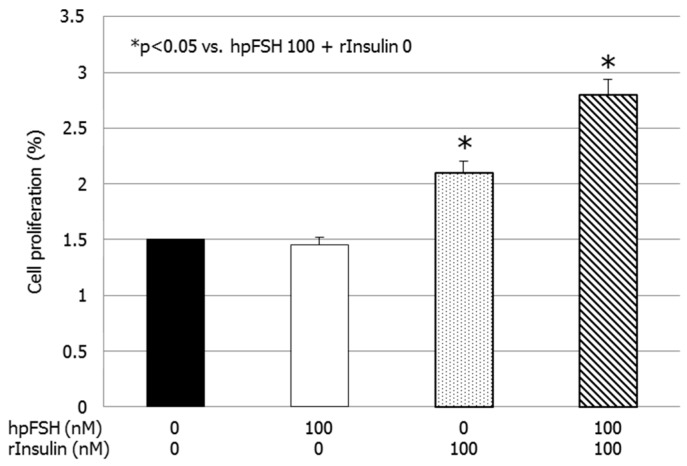
Cell proliferation assay. Data represent the mean ± SEM of three independent experiments, each performed in triplicate. * *p* < 0.05 vs. control (one-way ANOVA).

**Figure 5:**
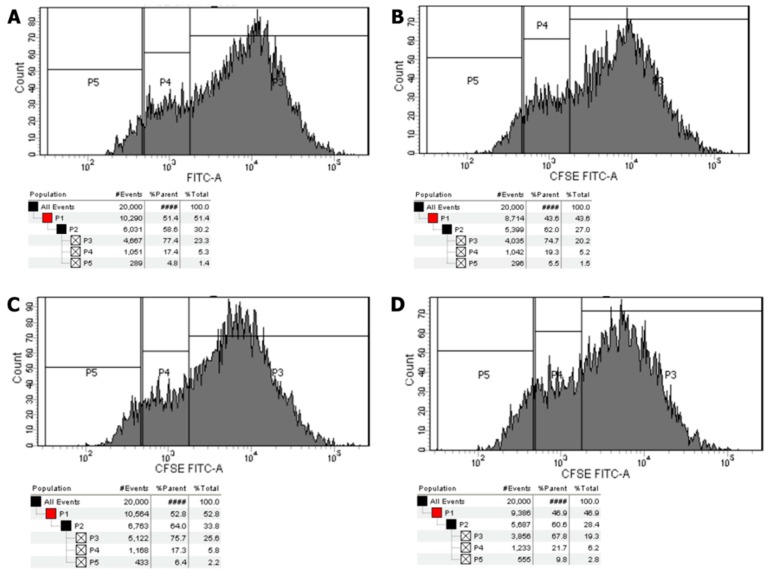
Flow cytometric analysis. Flow cytometric analysis of Sertoli cells stained with carboxyfluorescein diacetate N-succinmidyl ester (CFSE) without stimulation (**A**), and after incubation with hpFSH (**B**), rInsulin (**C**), hpFSH and rInsulin (**D**). Gray peaks represent successive generations.

Compared to the untreated control, the percentage of divided cells did not differ after exposure to hpFSH (1.5 ± 0.7 vs. 1.5 ± 0.1%, *p* > 0.99). Treatment with rInsulin or hpFSH plus rInsulin significantly increased the percentage of divided cells compared to the control (2.1 ± 0.1 vs. 1.5 ± 0.1%, *p* < 0.05 and 2.8 ± 0.0 vs. 1.5 ± 0.1%, *p* < 0.001) (Figure 4 and Figure 5).

## 4. Discussion

Metabolic diseases (e.g., obesity, insulin resistance, diabetes mellitus) have long been considered possible etiopathogenetic causes of male infertility [23,24,25]. However, the pathophysiological mechanisms are still elusive. To the best of our knowledge, no evidence is available on the effects of insulin on SC function. Our data suggest that SCs are responsive to insulin, which influences SC secretion patterns and proliferation. Similar to FSH, insulin downregulated AMH expression and secretion. Co-incubation with FSH and insulin lowered AMH secretion more than FSH alone. Furthermore, we found that insulin did not influence inhibin B expression and secretion, but FSH plus insulin lessened the effects of FSH, since inhibin B levels were lower in co-incubated cultures compared with those found in cultures stimulated with FSH alone.

These findings are consistent with the data reported in humans. An observational study carried out in pre-pubertal boys aged 5–9 years and young adult men aged 18–24 years found lower levels of inhibin B in obese (body mass index (BMI) > 30 kg/m^2^) patients compared to the normal weight (BMI < 25 kg/m^2^) controls. No difference was found in pre-pubertal boys. Unfortunately, despite the occurrence of hyperinsulinemia that might be supposed in the obese patients, insulin serum levels were not measured or reported [13].

More recently, a study involving 121 obese and 38 lean adolescents in the pubertal phase (Tanner stage ≥ 2) found lower levels of AMH and inhibin B in obese patients compared to controls. Obese patients also had higher levels of insulin whereas no significant differences in FSH levels were found [14]. It is noteworthy that the longitudinal study of the Western Australian Pregnancy Cohort (Raine), involving male children born in 1989–1991 followed from birth until the age of 20 years, has shown that patients with insulin resistance (Homeostatic Model Assessment of Insulin Resistance (HOMA) > 4), probably being hyperinsulinemics, had lower inhibin B and higher FSH levels at the age of 17–20 years, even after adjusting for age, body mass index, abstinence, history of cryptorchidism, varicocele, cigarette smoking, alcohol consumption and drugs (Hart et al., 2019). Low levels of AMH and inhibin B have been considered as biochemical signs of SC dysfunction in childhood and adolescence. Therefore, their measurement can help in the early identification of an isolated primary testicular tubulopathy, intercepting patients at risk of future infertility [4].

Altogether, these data highlight the negative impact of metabolic diseases on SC function, both before puberty, when only AMH secretion is affected, and after puberty, when inhibin B secretion is also impaired [13,14,15]. This is consistent with the results of the current study. SCs not exposed to FSH resemble a condition that occurs in pre-pubertal testes that are not physiologically exposed to FSH. At this time, hyperinsulinemia only affects AMH levels and does not have any effect on inhibin B [13,14]. Cells incubated with FSH provide data on how SCs can behave in the pubertal phase when they begin to be under the influence of FSH. In this phase of life, as suggested by human studies [13,14] and confirmed by the present in vitro data, hyperinsulinemia suppresses FSH-stimulated inhibin B secretion.

In addition, Hart and colleagues [15] observed lower sperm production in insulin-resistant 20 year old patients than in age-matched lean controls. Taken together, these data suggest a causal link between the increased prevalence of metabolic diseases [12] and the decline in sperm production [11]. Therefore, weight loss should be encouraged in obese children to preserve their future fertility.

The results of the present study suggest that a cross-talk between FSH and insulin might exist in porcine SCs. FSH has recently been shown to enhance myosin-phosphatase 1 (MYPT1), ERK 1/2, AKT^308^, AKT^473^ and to decrease JNK phosphorylation rates. These effects were partially or totally hindered by pre-treatment with the insulin-like growth factor 1 receptor (IGF1R) inhibitor NVP-AEW541, thus suggesting a role for IGF1R in FSH signaling [7]. Studies carried out in granulosa cells supported these data [26] and further indicated that insulin-receptor substrate 1 (IRS1) might be the hub linking FSHR, belonging to the G protein-coupled receptors (GPCRs), and IGF1R, which is a tyrosine kinase receptor [27]. IRS1 is involved in the insulin receptor signaling and is known to play a role in insulin sensitivity as insulin-resistant animal models show higher phosphorylation rates of this protein compared to healthy ones [28]. Taking all this into account, the cross-talk between FSH and insulin might involve IRS1, especially in case of insulin resistance, but this needs to be elucidated. Taking into account the findings of *FSHR* gene expression, we hypothesize that insulin might interfere with FSH signaling and action (which is consistent with data from insulin-resistant or obese children and adolescents) through *FSHR* downregulation. However, further studies analyzing the FSH and insulin receptors signaling pathway in SCs and the FSHR protein expression after hormone incubation are warranted to better clarify this topic.

We found an increase in AKT phosphorylation in SCs incubated with insulin. As mentioned before, the MAPK pathway is committed to cell proliferation [8]. As a result, and in contrast to FSH, insulin increased SC proliferation. This is in line with data on SC-selective INSR knock-out mice that have a lower testicular volume than that found in the wild type [10]. Data from a chicken model confirm the stimulating role of insulin on SC cultures [29]. These authors also reported a lack of AKT phosphorylation and SC proliferation in metformin-exposed SC cultures from insulin-resistant chickens [29]. The negative impact of metformin on SC proliferation was also confirmed in rats [30] and, consequently, the in vivo administration of metformin to pregnant mice caused the birth of pups with low testicular volumes, which was associated with a lower number of SCs [31]. Taken together, these data discourage metformin administration in obese boys, where SC is still actively proliferating [4]. The mechanisms through which this drug exerts such an anti-proliferative effect on SCs deserves further investigation.

Since the in vitro experimental model used in this study does not resemble the complexity of in vivo testicular tissue, the present findings should be taken with caution. We used a pure culture of SCs, and possible different in vivo responses due to the paracrine cross-talk between SC and Leydig cells cannot be excluded. Therefore, in vivo studies are needed to confirm such findings.

## 5. Conclusions

In conclusion, to our knowledge, this is the first study showing the influence of insulin on the secretion of AMH and inhibin B both basally and in response to FSH in primary porcine SC cultures. Under basal conditions, insulin suppressed AMH release but had no effect on inhibin B secretion. In addition, insulin influenced the SC responsiveness to FSH by lowering the amount of AMH and inhibin B compared to the cultures stimulated with FSH alone. Finally, insulin increased SC proliferation, confirming the results on mice with an SC-selective *INSR* gene knock-out [10]. These results could provide the rationale for the lower AMH and inhibin B levels found in obese or insulin-resistant boys and young men compared to normal weight controls [13,14,15], thus highlighting the importance of assessing testicular function in pre-pubertal obese children [10].
